# Analgesic Efficacy of Epidural Neuroplasty via Racz Catheter During Lumbar Fixation In Situ for Lumbosacral Spondylolisthesis: A Randomized Controlled Trial

**DOI:** 10.1155/anrp/1031307

**Published:** 2026-01-06

**Authors:** Ahmed Shehata Abd Elhamid, Mohammed Said Elsharkawy, Mostafa Mohamed Shaheen, Khaled Hamama, Ahmed Nada, Taysser M. Abdelraheem

**Affiliations:** ^1^ Anesthesia and Intensive Care Department, Faculty of Medicine, Port Said University, Port Said, Egypt, psu.edu.eg; ^2^ Anesthesiology, Surgical Intensive Care and Pain Medicine Department, Faculty of Medicine, Tanta University, Tanta, Egypt, tanta.edu.eg; ^3^ Neurosurgery and Neurointerventional Surgery Department, Faculty of Medicine, Port Said University, Port Said, Egypt, psu.edu.eg

**Keywords:** epidural neuroplasty, lumbar fixation, lumbosacral, pain, Racz catheter, spondylolisthesis

## Abstract

**Background:**

Effective pain management after lumbar fixation in situ is crucial for improving recovery and quality of life. Epidural neuroplasty via the Racz catheter is a potential method to enhance analgesia by targeting epidural inflammation and nerve compression. This work assessed the analgesic efficacy of epidural neuroplasty via a Racz catheter during lumbar fixation in situ for lumbosacral spondylolisthesis.

**Methods:**

This randomized, double‐blinded, controlled study was conducted on 50 patients aged 18–65 years, of both sexes, who had Grade 1 spondylolisthesis, facet osteoarthropathy, and a small disc on radiological findings. Participants were randomly assigned to two equal groups. Group S received epidural neuroplasty via a Racz catheter during lumbar fixation, and Group C received conventional lumbar steroid injections.

**Results:**

Visual analog scale and Oswestry low back disability questionnaire scores were significantly diminished immediately postprocedure and at 1, 2, 4, and 6 m in Group S than in Group C (*p* < 0.05). Hypotension, paraesthesia, bleeding, and headache exhibited comparability between the two groups. The patient satisfaction level was significantly elevated in the Racz catheter group as opposed to the conventional lumbar group (*p* < 0.05).

**Conclusions:**

Epidural neuroplasty using a Racz catheter during lumbar fixation provides enhanced short‐term analgesia, functional recovery, and patient satisfaction compared with conventional lumbar treatment in Grade‐1 spondylolisthesis, without increased adverse effects, providing preliminary evidence that warrants validation in larger, long‐term studies.

**Trial Registration:** ClinicalTrials.gov identifier: NCT06684821

## 1. Introduction

Spondylolisthesis is a relatively common spinal condition that is classified into five main types according to the Wiltse classification system: dysplastic, isthmic, degenerative, traumatic, and pathologic [1]. Among these, lumbosacral spondylolisthesis is particularly prevalent. It involves the forward slipping of one vertebra over the one beneath it, often leading to chronic back pain and a variety of neurological symptoms. This condition may significantly affect a patient’s mobility and overall quality of life, making it a major contributor to spinal morbidity [2].

Despite its potential impact, many people with spondylolisthesis are asymptomatic. In fact, it is often detected incidentally during routine X‐rays. Symptomatic patients, however, typically seek medical care because certain activities aggravate their low back pain (LBP) [3]. LBP, defined as pain localized between the lower rib cage and the gluteal folds, may occur alone or with radiating leg pain, further complicating diagnosis and treatment [4].

LBP remains one of the leading causes of chronic pain and disability worldwide. It is often labeled as lumbosacral radicular syndrome, lumbar radiculopathy, nerve root pain, or nerve root irritation. Frequently, the pain starts in the lumbar spine and radiates down the legs, causing additional functional impairment [5].

For most patients with spondylolisthesis, conservative treatment is the primary line of management, regardless of whether neurological symptoms are present. Pain is usually controlled through pharmacological options like nonsteroidal anti‐inflammatory drugs (NSAIDs) and other analgesics. Physical therapy interventions, including bracing and flexion‐based strengthening exercises, have also shown considerable benefits. When conservative treatments are not enough to relieve symptoms, epidural steroid injections are often used to help control inflammation and manage pain [6, 7]. Surgical lumbar fixation remains a widely accepted option for more serious cases, especially those resulting from trauma, pathological changes, or degenerative conditions of the spine [8].

In cases of chronic LBP unresponsive to medical therapy, epidural lysis of adhesions offers another treatment option [9]. The Racz approach, as delineated by Racz et al. [10]. It involves a combination of epidurography and the injection of hyaluronidase, hypertonic (10%) saline, and bupivacaine. In this method, the catheter is left in place for 3 days, with additional injections of hypertonic saline and bupivacaine administered on the second and third days [11].

However, research on combining epidural neuroplasty with a Racz catheter and in situ lumbar fixation to manage lumbosacral spondylolisthesis remains limited. This study was designed to address that gap, aiming to assess the analgesic effectiveness of epidural neuroplasty with a Racz catheter during lumbar fixation in patients with lumbosacral spondylolisthesis.

## 2. Patients and Methods

This randomized, controlled, double‐blind study included 50 patients (both men and women) aged 18–65 who were diagnosed with Grade 1 spondylolisthesis and facet joint osteoarthropathy, with minor disc abnormalities identified on imaging.

This study was approved by the institutional Ethics Committee (No.: 36264PR903/10/24) from September 2024 to February 2025. The study procedures follow the guidelines outlined in the World Medical Association (WMA) Declaration of Helsinki, and written informed consent was obtained from all subjects participating in the trial or their next of kin before study commencement.

Exclusion criteria ruled out uncooperative patients, those who required discectomy for lumbar disc herniation, and individuals with severe spinal canal stenosis needing laminectomy. Other exclusions included patients with coagulopathy, active skin infections, a BMI of 35 kg/m^2^ or greater, known allergies to contrast agents, or a history of prior conventional spinal surgery.

Before surgery, all patients underwent a thorough evaluation, which included a complete medical history, a clinical examination, and any necessary laboratory tests. The diagnosis and affected level (specifically L5–S1) were confirmed by clinical assessment and imaging, including lumbar MRI and X‐rays. Patients were also carefully instructed on how to use the visual analog scale (VAS) to report their pain levels accurately.

### 2.1. Randomization

To ensure a precise study design, randomization was performed using a computer‐generated randomization scheme. Each patient received a unique code placed in a sealed, opaque envelope to maintain allocation concealment and minimize selection bias. Patients were randomly assigned in equal numbers to one of two groups: Group S, who underwent epidural neuroplasty with a Racz catheter during lumbar fixation, and Group C, who received conventional lumbar epidural steroid injections.

Operator blinding was impracticable owing to procedural discrepancies. Nonetheless, both patients and outcome assessors were blinded to group allocation. All procedures were conducted under the same sterile conditions, and identical postoperative dressings were utilized to maintain blinding. Outcome assessments were performed by independent, blinded assessors who were uninvolved in the processes and unaware of group allocations, thereby minimizing assessment bias.

All participants underwent standardized preoperative preparation, including the placement of a 20‐gauge (20G) peripheral intravenous (IV) cannula for vascular access. Intraoperative monitoring included capnography (end‐tidal CO_2_), pulse oximetry, noninvasive blood pressure (NIBP), electrocardiography (ECG), and body temperature monitoring. To reduce the risk of postoperative infection, all patients received a prophylactic IV dose of 1 g of cefazolin before the procedure [11].

### 2.2. Classic Lumbar Fixation Technique

The lumbar fixation procedure was performed through a posterior midline incision, which allowed direct access to the L5–S1 segment. After careful subperiosteal dissection, the exposure was extended to the tips of the L5 transverse processes, the L5/S1 facet joints, and the sacral ala. Stabilization was achieved by inserting segmental pedicle screws into the L5 and S1 vertebrae, providing robust structural support to the spine.

### 2.3. Epidural Neuroplasty Racz Catheter Technique

The patient was positioned prone in the operating room, with a cushion under the belly to rectify lumbar lordosis and an additional pillow under the ankles for comfort. Following sterile preparation, the sacral hiatus was located by palpation slightly inferior to the sacral cornu or with fluoroscopic assistance. A local anesthetic was administered subcutaneously one inch laterally and two inches caudally from the sacral hiatus.

A 16‐gauge Racz needle (Epimed International, Inc., Johnstown, NY) was inserted into the skin at a 45° angle under fluoroscopic guidance. Upon the needle traversing the hiatus, the angle was calibrated at 30°, and accurate positioning of the needle inside the epidural space was verified. Following negative aspiration, 2 mL of Omnipaque (GE Healthcare [Shanghai] Co., Ltd., Shanghai, China) was administered through the needle, facilitating visualization of contrast dispersion (Figure 1).

Figure 1Racz catheter after lumbar fixation.

**Figure:**
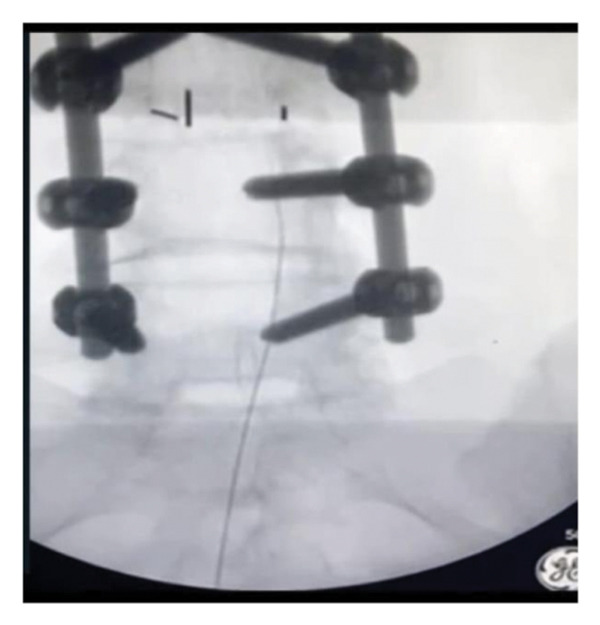
(a)

**Figure:**
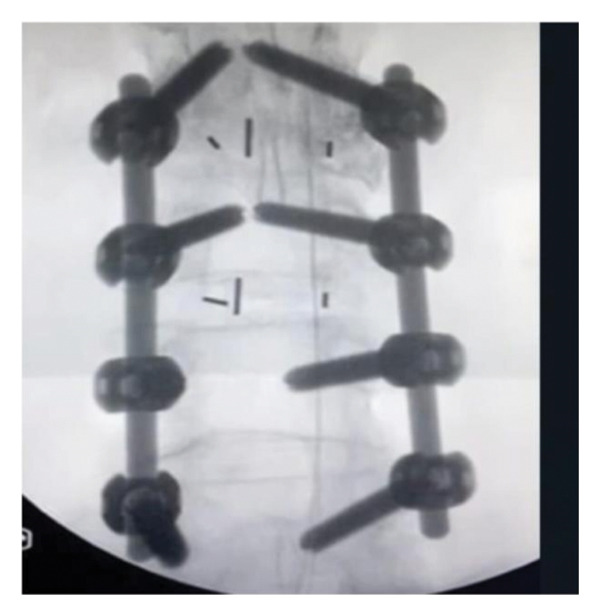
(b)

### 2.4. Conventional Epidural Steroid Injections

In Group C, patients received conventional epidural steroid injections before the fixation procedure. In the prone position, the sacral hiatus was located via palpation or fluoroscopic guidance. An 18‐gauge Tuohy epidural needle (PAJUNK GmbH, Germany) was introduced into the epidural space employing the loss‐of‐resistance technique under fluoroscopy. After confirming proper epidural placement with negative aspiration and contrast injection, a steroid solution comprising 80 mg of methylprednisolone acetate mixed with normal saline (total volume of 8–10 mL) was slowly injected into the epidural space at the L5‐S1 level to alleviate inflammation and pain (Figure 2).

Figure 2Epidural (a) AP view and (b) lateral view after contrast administration.

**Figure:**
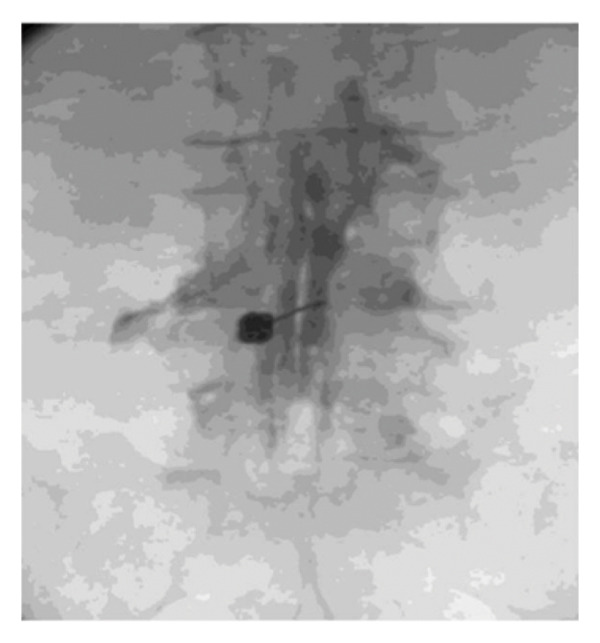
(a)

**Figure:**
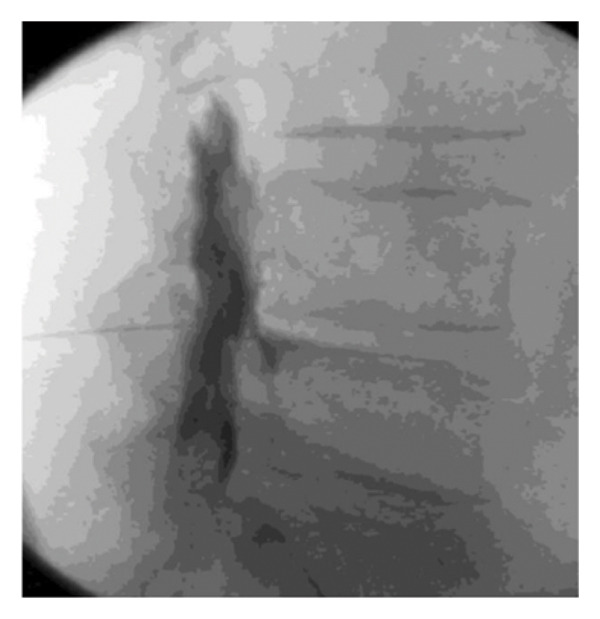
(b)

### 2.5. Postoperative Care

Postoperative care included a structured pharmacological regimen for pain management and infection prevention. This comprised oral antibiotics for five days, intramuscular diclofenac sodium (75 mg every 12 h for one week) for analgesia, gastric protectants (omeprazole 20 mg orally, every 12 h for one week) to prevent NSAID‐induced gastric irritation, and muscle relaxants (baclofen 10 mg orally, every 12 h) to alleviate muscle spasms. A multimodal analgesic protocol was implemented, including paracetamol (1 g every 6 h) for baseline pain control. Rescue analgesia with morphine was administered as a 3 mg bolus when the VAS score exceeded 3, with additional doses given every 30 min as needed until the VAS score fell below 4. VAS assessments were conducted preoperatively, immediately postoperatively, and at 1, 2, 4, and 6 months postprocedure [12].

To evaluate functional disability, we used the Oswestry low back disability questionnaire (OSW), a well‐established, validated tool that assesses how LBP affects daily activities. The questionnaire consists of 10 items, each scored from 0 to 4. Based on the total score, patients are classified into the following categories: 0–4 (no disability), 5–14 (mild disability), 15–24 (moderate disability), 25–34 (severe disability), and 35–50 (complete disability). We first administered the questionnaire before surgery to set a baseline, and then repeated it at 1, 2, 4, and 6 months after the procedure [13].

Patient satisfaction was gauged separately via a simple 5‐point Likert scale, where one meant “extremely dissatisfied” and five meant “extremely satisfied” [14].

To assess the efficiency of the procedures, we recorded the duration of each technique in minutes. We also carefully documented any side effects that occurred during or after the interventions.

The primary measure for the study was the change in Oswestry Disability Index (ODI) scores. Secondary outcomes included changes in pain intensity, as measured by VAS scores, patient satisfaction levels, time per procedure, and procedural complications.

### 2.6. Sample Size Calculation

We determined the necessary sample size via G∗Power software, version 3.1.9.2 (University of Kiel, Germany), which is specifically designed for statistical power analysis. To ensure accuracy, we first conducted a small pilot study with 5 participants per treatment group. Results from the pilot indicated that the mean ODI score for patients who underwent the conventional lumbar epidural technique was 25.6 ± 13.67, while it was 14.2 ± 7.66 for those treated with the Racz catheter. Based on these preliminary findings, the sample size was calculated at an effect size of 1.029, with 95% confidence and 90% power. We maintained a 1:1 group ratio to ensure balanced comparisons. To anticipate potential dropouts or losses during follow‐up, we added four additional participants to each group. This brought the final target to 25 patients per group, ensuring we had sufficient data for robust statistical analysis and reliable conclusions.

### 2.7. Statistical Analysis

All statistical analyses were performed via SPSS software, version 27 (IBM, Armonk, NY, USA). The normality of the data distribution was evaluated via the Shapiro–Wilk test, and a graphical assessment was conducted by reviewing histograms. For parametric quantitative variables, data were expressed as mean ± standard deviation (SD) and analyzed using an unpaired Student’s *t*‐test to compare group differences. In contrast, nonparametric quantitative variables were represented as medians and interquartile ranges (IQRs) and assessed via the Mann–Whitney test to account for data that did not meet normality assumptions. Categorical data were summarized as frequencies and percentages and evaluated via either the chi‐square test or Fisher’s exact test, depending on the expected frequency distribution. A two‐tailed statistical approach was employed to ensure rigorous analysis, with a significance threshold set at *p* < 0.05. All randomized patients were analyzed according to assigned groups (intention‐to‐treat).

## 3. Results

**Figure 3:**
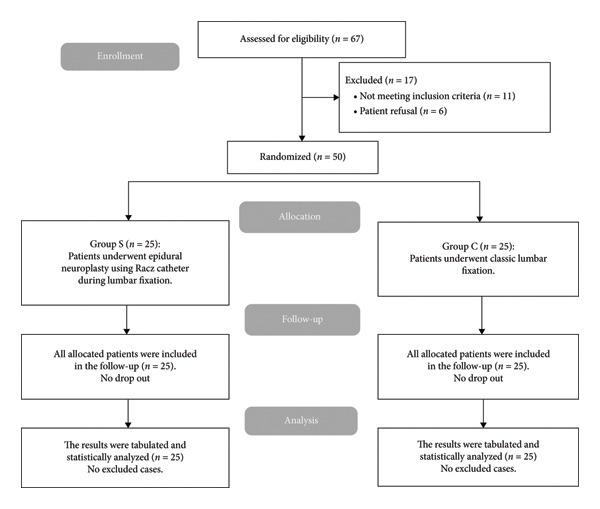
CONSORT flowchart of the enrolled patients.

Figure 3 illustrates that 67 cases were enrolled, and their eligibility for participation was assessed. 11 patients did not meet the inclusion criteria. Six participants refused to participate, resulting in 50 cases randomized to two groups for subsequent analysis.

Table 1 indicates that demographic data and procedure time were similar across the cohorts.

**Table 1:** Demographic data and procedure time of the studied groups.

	Group S (*n* = 25)	Group C (*n* = 25)	*p*
Age (years)	43.32 ± 11.96	40.6 ± 11.92	0.425
Sex			
Male	15 (60%)	17 (56.67%)	0.258
Female	10 (40%)	13 (43.33%)	
Weight (kg)	80.6 ± 7.63	83.48 ± 7.12	0.174
Height (cm)	167.76 ± 7.11	168.6 ± 7.1	0.678
BMI (kg/m^2^)	28.71 ± 2.92	29.48 ± 3.19	0.376
Procedure time (min)	56.2 ± 7.4	53.6 ± 10.66	0.321

*Note:* Data are presented as mean ± SD or frequency (%).

Abbreviation: BMI, body mass index.

Table 2 indicates that the VAS score was not significantly different at baseline between the two groups. However, it was significantly diminished immediately postprocedure and at 1, 2, 4, and 6 m in Group S than in Group C (*p* < 0.05).

**Table 2:** VAS of the studied groups.

	Group S (*n* = 25)	Group C (*n* = 25)	*p*
Baseline	7 (6–8)	6 (6–7)	0.390
	6.68 ± 1.03	6.44 ± 1.23	

Immediate postprocedure	5 (4–5)	6 (5–7)	0.007^∗^
	4.76 ± 1.09	5.8 ± 1.38	

1 m	7 (6–8)	6 (6–7)	0.003^∗^
	3.8 ± 1.12	4.96 ± 1.24	

2 m	5 (4–6)	6 (5–7)	0.002^∗^
	2.76 ± 1.2	3.92 ± 1.08	

4 m	2 (2–3)	3 (3–4)	0.001^∗^
	2.28 ± 0.79	3.16 ± 0.94	

6 m	2 (1–2)	2 (2–3)	0.021^∗^
	1.96 ± 0.84	2.6 ± 1	

*Note:* Data are presented as median (IQR) or mean ± SD.

Abbreviation: VAS, visual analog scale.

^∗^Significantly as *p* ≤ 0.05.

Table 3 indicates that the OSW score did not differ significantly at baseline between the two groups. However, it was significantly diminished immediately postprocedure and at 1, 2, 4, and 6 m in Group S than in Group C (*p* < 0.05).

**Table 3:** OSW of the studied groups.

	Group S (*n* = 25)	Group C (*n* = 25)	*p*
Baseline	23 (17–25)	21 (18–26)	0.755
	21.24 ± 6.36	21.88 ± 4.03	

Immediate postprocedure	15 (9–19)	16 (15–21)	0.037^∗^
	13.6 ± 6.87	17.96 ± 4.29	

1 m	9 (3–15)	13 (9–16)	0.019^∗^
	8.84 ± 5.93	13.04 ± 4.73	

2 m	6 (1–11)	11 (7–14)	0.019^∗^
	6.76 ± 4.96	10.44 ± 4.6	

4 m	5 (1–7)	7 (4–10)	0.035^∗^
	4.88 ± 3.64	7.32 ± 3.78	

6 m	3 (1–4)	7 (3–8)	0.009^∗^
	3.2 ± 2.2	5.92 ± 3.8	

*Note:* Data are presented as median (IQR) or mean ± SD. OSW, Oswestry low back disability questionnaire.

^∗^Significantly as *p* ≤ 0.05.

Table 4 indicates that hypotension, paresthesia, bleeding, and headache were insignificantly different between the two groups. Bending of the needle tip, shearing of the catheter, migration of the catheter, misplacement of the catheter, blocking of the catheter, and infection did not occur in any patient in either group. Patient satisfaction was significantly higher in Group S than in Group C (*p* < 0.05).

**Table 4:** Side effects and patients’ satisfaction of the studied groups.

	Group S (*n* = 25)	Group C (*n* = 25)	*p*
*Side effects*			
Bending of the tip of the needle	0 (0%)	0 (0%)	—
Shearing of the catheter	0 (0%)	0 (0%)	—
Blocking of the catheter	0 (0%)	0 (0%)	—
Misplacement of the catheter	0 (0%)	0 (0%)	—
Migration of the catheter	0 (0%)	0 (0%)	—
Blood aspiration	0 (0%)	0 (0%)	—
Hypotension	1 (4%)	3 (12%)	0.609
Paresthesia	0 (0%)	1 (4%)	1
Bleeding	0 (0%)	2 (8%)	0.489
Headache	1 (4%)	0 (0%)	1
Infection	0 (0%)	0 (0%)	—

*Patients’ satisfaction*			
Extremely satisfied	16 (64%)	7 (28%)	0.038^∗^
Satisfied	6 (24%)	7 (28%)	
Neutral	2 (8%)	9 (36%)	
Unsatisfied	1 (4%)	2 (8%)	
Extremely dissatisfied	0 (0%)	0 (0%)	

*Note:* Data are presented as frequency (%).

^∗^Significantly as *p* ≤ 0.05.

## 4. Discussion

The approaches employed in chronic lumbar radicular pain management encompass pharmacological analgesics, centrally acting agents, lumbar epidural and transforaminal epidural steroid injections, physical therapy and rehabilitation, dorsal root ganglion pulse radiofrequency, and epidural lysis [15].

Several investigations have indicated that epidural neuroplasty may cure chronic LBP [16–18]. Percutaneous epidural neuroplasty (PEN) is often used in patients with chronic LBP or those who have not had improvement after back surgery syndrome [19].

We chose conventional lumbar epidural steroid injection as the control because it epitomizes the typical perioperative analgesic protocol for lumbosacral radicular pain [20]. A fixation‐only group may more effectively isolate the impact of Racz neuroplasty; nevertheless, comparison with the existing standard more accurately represents clinical reality and assesses its supplementary advantages [21].

This study found a significantly diminished OSW (immediately postoperative and at 1, 2, 4, and 6 months) in the Racz group than in the conventional lumbar group (13.6 ± 6.87 vs. 17.96 ± 4.29, 8.84 ± 5.93 vs 13.04 ± 4.73, 6.76 ± 4.96 vs. 10.44 ± 4.6, 4.88 ± 3.64 vs. 7.32 ± 3.78, and 7.32 ± 3.78 vs. 7.32 ± 3.78, respectively). We observed that postoperative pain (immediately postoperative and at 1, 2, 4, and 6 months) was significantly improved in the Racz group compared to the conventional lumbar group. The safety profiles of both techniques were comparable, with similar incidences of side effects (hypotension, paresthesia, headache, and infection). Patients in the Racz group had higher satisfaction rates than those in the conventional lumbar group.

In agreement with our findings, Ege [22] found significant decreases in ODI scores at 1 and 6 months after epidural neuroplasty via Racz catheter, confirming our findings (*p* < 0.001). They found that epidural neuroplasty employing the Racz catheter resulted in considerably lower pain levels after one and 6 months compared to before treatment (*p* < 0.001).

In line with our results, Karm and colleagues [18] evaluated the effectiveness of PEN via a balloon (Racz) catheter in patients with lumbar spinal stenosis and degenerative lumbar spondylolisthesis. They concluded that PEN utilizing a balloon catheter may be an alternative treatment option for patients with chronic LSS, regardless of accompanying DLS, who have failed conservative management.

Moreover, Choi et al. [17] compared the 6 month outcomes of endoscopic epidural neuroplasty and PEN in LBP. They noted that ODI scores (at 1 and 6 m) decreased significantly from pre‐PEN levels (31.7 ± 3.6, 27.8 ± 1.8, and 26.9 ± 2.3, respectively). They showed that pain scores (at 1 and 6 m) decreased significantly compared to pre‐PEN (6.5 ± 0.8, 2.3 ± 0.7, 4.6 ± 1.0, and 4.3 ± 0.7, respectively).

Also, Ji et al. [23] stated that targeted Racz catheter‐based neuroplasty provided superior clinical outcomes compared to a cervical epidural steroid injection, demonstrating better functional recovery and greater pain reduction. However, they found a significant difference in disability scores only at 6 months, with no significant differences at 2, 4, and 12 months.

Additionally, Moon and colleagues [24] exhibited that epidural neuroplasty via a Racz catheter significantly improved pain and functional outcomes in patients with cervical spinal pain, with sustained benefits up to 12 months.

Moreover, a systematic review and meta‐analysis by Park and co‐authors [25] reported that at 3 months, the mean difference in pain reduction favoring epidural neuroplasty over pulsed dorsal root ganglion radiofrequency was −1.47 (95% CI: −2.73 to −0.46), and both approaches exhibited comparability at 1 month.

Also, Kim and collaborators [26] reported a pooled complication rate of 9.0% (95% CI: 6.7%–12.1%), with most adverse events being minor and transient, such as temporary paresthesia or catheter‐related mechanical issues, while serious complications were rare.

The comparable safety profile between epidural neuroplasty via a Racz catheter and classic lumbar fixation may be attributed to several factors. Both procedures are considered minimally invasive compared to open surgical techniques. While lumbar fixation involves surgical stabilization of the spine, epidural neuroplasty via a Racz catheter is typically performed percutaneously. This reduces the overall risk factors associated with more invasive surgeries [27].

The research is limited by a relatively small sample size and a single‐center design. Short‐term follow‐up (6 m) may not fully capture long‐term efficacy or pain recurrence, as the effects of Racz catheter neuroplasty could diminish over time, and this timeframe does not allow conclusions regarding treatment durability. Future studies with extended follow‐up are recommended to assess the durability of these outcomes. Larger‐scale studies with larger sample sizes could help confirm these findings and provide a more robust assessment of the efficacy and safety of epidural neuroplasty across different patient subgroups with varying grades of spondylolisthesis. A longer follow‐up would be valuable for capturing the long‐term effects of neuroplasty on pain and function.

## 5. Conclusions

Epidural neuroplasty using a Racz catheter during lumbar fixation provides enhanced short‐term analgesia, functional recovery, and patient satisfaction compared with conventional lumbar treatment in Grade‐1 spondylolisthesis, without increased adverse effects, providing preliminary evidence that warrants validation in larger, long‐term studies.

## Ethics Statement

This study was approved by the Institutional Ethics Committee (Approval No. 36264PR903/10/24). Written informed consent was obtained from all participants (or their legally authorized representatives) after a verbal and written explanation of the study aims, randomization procedures, risks, benefits, alternatives, confidentiality, and the right to withdraw at any time. All postrandomization withdrawals and losses to follow‐up were recorded, along with the reasons, and are shown in the CONSORT flow diagram.

## Disclosure

All authors commented on previous versions of the manuscript. All authors read and approved the final manuscript.

## Conflicts of Interest

The authors declare no conflicts of interest.

## Author Contributions

All authors contributed to the conception and design of the study. Material preparation, data collection, and analysis were performed by Mohammed Said Elsharkawy, Taysser M. Abdelraheem, and Ahmed Shehata Abd Elhamid. The first draft of the manuscript was written by Mostafa Mohamed Shaheen, Khaled Hamama, Ahmed Nada, and Ahmed Shehata Abd Elhamid.

## Funding

No funding was received for conducting this study.

## Data Availability

Data are available on request from the authors.
